# Supplementary material for: Distributional effects of parental time investments on children’s socioemotional skills and nutritional health
Source: PLoS One. 2023 Oct 13;18(10):e0288186. doi: 10.1371/journal.pone.0288186 (PMC10575499; doi:10.1371/journal.pone.0288186)
Supplement: S1 Appendix — (PDF) [file pone.0288186.s001.pdf]

S1 Appendix. National Board of School Aid and Scholarships

Chile has several long-standing social programs directed to children and their families in the school context. Since 1964, the National Board of School Aid and Scholarships (JUNAEB, Spanish acronym), an agency part of the Ministry of Education, has been responsible for assessing students' needs and allocating resources through different programs. Their mission statement follows[1]:

> *To support all students in a condition of social, economic, psychological and/or biological disadvantage, by providing quality, comprehensive products and services, that contribute to the realization of equal opportunities, human development and social mobility.*

JUNAEB manages programs and services covering all educational levels from pre-school to college. The range of programs includes: medical and dental services, nutrition, stimulation and mental health, scholarships, transport, housing and school supplies. The two largest programs within JUNAEB are the School Meals Program (since 1964) and the Abilities for Life Program, AfLP, (since 1999). Both programs are considered large relative to the served population (as a fraction of target students), in comparison to similar programs in other countries.

Since 2016, the SMP covers the 60% of students based on vulnerability at the individual level.[2] As of 2018, AfLP provided services to 30% of public and subsidized schools, targeted by the proportion of vulnerable students attending each school. Given eligibility, participation in the AfLP for schools (and their communities) is voluntary. During the last decades, both programs have provided support to hundreds of thousands of families with adequate nutrition and mental health services.

JUNAEB administrative data

Every year, JUNAEB requires the assistance of all schools participating in the SMP to collect a census on the health and vulnerability of children attending such schools (regardless of SMP eligibility). Children from pre-school, first, fifth and ninth grade participate in anthropometric measurements and their parents complete an extensive household and child survey. These two components form the Nutritional Map (NM) the Vulnerability Survey (VS). In 2015, 742,489 children had both instruments applied, this is 90% of all students attending public or private subsidised schools. The coverage of the instruments is remarkable, considering that average daily attendance rates in Chile, as well as many developed countries, is close to 90%. Annual reports from JUNAEB show that coverage rates for the instruments has not changed significantly over time.[3] As noted in section 3, I refer to SMP data as the dataset for the sub-sample of students with valid NP and VS instruments.

S2 Appendix Table 1 summarizes a comparison between official enrollment data and the population with SMP data in the 2014-2015 cohort.[4] Compared to Kindergarten, SMP data coverage is lower in first grade, which can be explained by two factors. First, While SMP in pre-school is virtually universal, several subsidized schools have no participation in the program, hence SMP data is not collected. Secondly, average daily attendance decreases as children move through the educational system.

[1]Translated from JUNAEB website

[2]Vulnerability and eligibility criteria is defined and measured as explained in Section 2.

[3]For more see JUNAEB Nutritional Map.

[4]Similar calculations for the 2012-2018 cohort are available upon request.

The NM is conducted by the class professor (or the professor designated by the school) through direct measurement of children's weight and height, as well as presence of cavities. While there is significant variation in the methods and instruments used for the measurements, the distribution of data is consistent across sub-populations and over time. Studies conducted in random samples of Chilean students show that while the distribution of measurements from teachers are not substantially different than trained professionals, there is room for missclassification of nutritional status due to noise introduced by variation in the methods and instruments used by teachers [?, ?]. Evidence suggests that teachers are more likely than trained professionals to heap (round) weight and height measures, which create important discrepancies in the BMI-z averages.

The VS contains rich information at the household level to characterize vulnerability along with several dimensions of child's health and development. The instrument presents some differences between each educational level. The common information is: household composition and interactions with index child, geographic location and cultural background, educational attainment and occupation of caregivers, physical resources for learning/development, children's health status and educational background. Also in all years there are questions regarding birth and breastfeeding frequency. There are two sections that are different between pre-school and the school years. The first one relates to paternal time investments (only available in pre-school) and the second one relates to social and emotional aspects the child (only available in school grades, with slight variation across grades).[5] VS data has been consistently collected and coded since 2007 (including the generation of standarized anthropometric measurements from the MN using 2007 WHO reference guide). However, there are two important caveats to constructing longitudinal information at the household level. First, the quality of the data in the year 2013 is limited due to changes in the questionnaire recording format, affecting all grades. Secondly, the surveys before and after 2015 contain slight variations in the context of the questionnaire. For example, a section on children health difficulties is only introduced from year 2014. As a result, for the 2014-2015 cohort, it is not possible to construct latent factors in both periods. Information on the effect that variation in the sections of the VS questionnaire affects the model specification in each cohort is explained in Appendix B.

S1 Appendix Table 1. School enrollment and SMP data

	Kindergarten 2014			1st Grade 2015		
	MINEDUC	JUNAEB		MINEDUC	JUNAEB	
Public and subsidized	193,713	188,512	97%	236,201	200,063	85%
Public	74,098	70,067	95%	94,152	85,082	90%
Subsidized	119,615	118,445	99%	142,049	114,965	81%

SMP: School Meal Program (JUNAEB).

[5] A version of the VS questionnaires (in Spanish) can be acquired from JUNAEB, upon request.
