# Supplementary material for: Distributional effects of parental time investments on children’s socioemotional skills and nutritional health
Source: PLoS One. 2023 Oct 13;18(10):e0288186. doi: 10.1371/journal.pone.0288186 (PMC10575499; doi:10.1371/journal.pone.0288186)

S2 Appendix. Socioemotional development and parental investments

In the last decade, several economists have provided a strong framework to incorporate psychological constructs into economic models. This framework is often referred as the production technology of early human capital (or SED). Although measuring cognition and physical development has been widely studied, less consensus exists on characterizing and measuring SED. A main issue is that SED can only be proxied. Psychology, neuroscience and similar fields provide strong theoretical background and extensive evidence on survey items and inventories that consistently identify a given personality (or character) construct. Personality constructs contain a mixture of two components: the part that is malleable over time and the portion that is mostly inheritable and stable in the life-cycle. Throughout this paper, I refer to SED as those that, at least to some extent, can be shaped during developmental stages. These SED can be considered equivalent to character constructs discussed in the psychology literature, such as personality traits.[6]

The extent that personality traits relate to behavior is part of a larger and complex system. As such, for any given level of personality traits, these can be interpreted as the anchor from which behavior varies depending on the situation. In the economic and psychology literature, several authors have model socioemotional development among children using these personality traits and other measures of behavioral performance (e.g. inhibitory control, executive functioning, resilience), as they are consistent with the definition of SED: malleable over time and predict relevant economic and social outcomes in the short and long term.

Current evidence from several programs and interventions at different ages elucidates a joint production of cognition, physical health and SED during early childhood. The link between physical health and cognition has been widely studied. The connection between socioemotional development and mental health in children (and adults) is less understood. While some personality traits have been associated with higher likelihood of mental disorders (depression, ADHD, addiction), neuroscience scholars are only beginning to study the biological basis of how cognition, personality, values, identity and memory direct behavior. Nevertheless, personality traits are consistent predictors of behavior and can be fostered during early childhood, thus being a policy-relevant starting point to study the connection between socioemotional development and specific health behaviors.

From an empirical perspective, consistently measuring SED relies in the psychometric properties of the questionnaires that are developed to elicit specific constructs. There is a myriad of different inventories and scales that capture different dimensions of personality, development and behavior. Some of this off-the-shelf questionnaires have been extensively studied in terms of their construct validity. However, in many cases, instead of relying on off-the-shelf surveys, programs and interventions develop their own ad-hoc questionnaires (e.g. Perry Program). Regardless, the same principles and methods for analysis of construct validity can be applied, in order to develop consistent measures of SED. In the remainder of this section I further describe the steps to obtain SED and parental investment factors from the items in the VS data.

[6] Some studies refer to these traits as the stable, inheritable part of personality. However, I avoid such distinction in order to remain consistent with the language used in economics and psychology

Measures available in the dataset

The VS in first grade has two sections where aspects of socioemotional and cognitive development are captured. The first set of questions document health-related behavioral difficulties, including motor, visual/hearing, self-control, learning and task performance. The second set measures aspects of affection, social interactions and curiosity.

An important feature of the proxy measures in the VS is the emergence of response styles, i.e., consistent patterns of response across items for each individual. In this case, a large fraction of parents have a tendency of consistently report "desirable" behavior from their children, alongside with minimal behavioral difficulties (13% of parents respond the lowest value on the scale to 20/22 items). Extensive literature proposed methods to address the presence of response styles when measuring personality constructs. I model response styles as individual (random) intercepts that are common across all measures. Another feature of the survey items on the VS data is how questions are framed to elicit a given response. All but one of the questions are phrased such that lower values are associated with desirable/healthy behavior.

Exploratory factor analysis

A starting point to characterize skill constructs is to conduct Exploratory Factor Analysis (EFA), to unveil the potential structure of the measurement system. I separately estimate the measurement system for SED and investments, for two reasons. First, a large fraction of students are not linked longitudinally, and excluding them from analysis can affect the underlying distribution of underlying factors. Secondly, while response styles are observed when parents respond to child's behavior, answers directed towards time investments do not present similar skewness. Thus, imposing a random intercept across all survey items would not be recommended.

S2 Apppendix Table 1 reports the rotated factor loadings from EFA with random intercepts. Most questions load into one factor, consistent with previous studies that propose a dedicated measurement system, i.e. each measurement loads into one factor. Many criteria have been proposed to determine the number of factors. Based on the questions' content and structure, as well as the rotated factor loadings, I consider three of the factors to be consistent with dimensions of analysis: Externalizing Behavior, parental time investment and a process measure of learning.

Confirmatory factor analysis

The next step is to estimate the dedicated measurement system, as presented in Methods section. The scale in all questions used to elicit socioemotional skill factors are inverted to facilitate interpretation. As discussed, I follow standard normalization of loadings and mean factors for identification, while introducing a random intercept across measurements to capture response styles. The measurement system is estimated by approximating the distribution of latent factors by mixture or joint normal distributions and allowing the error terms to be independent and normally distributed. Initially, the system was estimated allowing for different loading for each SMP eligibility group, however there are not statistically significant differences between eligibility groups and the factor loadings or mixture weights. Therefore, the final system is estimated assuming equal factor loadings across eligibility groups. S2 Appendix Fig 1 shows the density of the estimated random intercept. Most parents in the data express a significant response style that correlates positively with parent's education and expectations regarding their children's human capital attainment, which suggests social desirability bias.

S2 Appendix Table 1. Quatimin-rotated factor loadings (random intercept EFA, standarized values)

Measurements	θ^O		θ^E		θ^N		L	
	Factors							
difficult to perform a task	-0.014	*0.001*	0.028	*0.001*	-0.014	*0.002*	**0.920**	*0.002*
difficult to complete homework	-0.008	*0.001*	0.026	*0.001*	0.007	*0.002*	**0.904**	*0.002*
difficult to understand others	0.125	*0.006*	-0.096	*0.006*	**0.313**	*0.007*	0.255	*0.006*
difficult to learn	0.161	*0.005*	-0.108	*0.005*	0.212	*0.006*	**0.495**	*0.006*
difficult to control behavior	0.027	*0.003*	-0.052	*0.003*	**0.678**	*0.007*	0.127	*0.007*
difficult to get along with peers	-0.041	*0.003*	0.108	*0.005*	**0.686**	*0.004*	-0.058	*0.002*
affection to family	0.034	*0.005*	**0.580**	*0.006*	-0.005	*0.004*	0.022	*0.003*
affection to peers	-0.012	*0.005*	**0.632**	*0.006*	0.132	*0.005*	-0.002	*0.003*
express feelings	0.025	*0.005*	**0.638**	*0.006*	-0.081	*0.003*	0.059	*0.003*
shows feeligs phisically	0.030	*0.005*	**0.687**	*0.006*	-0.043	*0.003*	0.042	*0.002*
plays with peers	0.102	*0.008*	**0.458**	*0.009*	0.147	*0.007*	-0.056	*0.005*
shares with peers	0.116	*0.007*	**0.353**	*0.008*	0.208	*0.006*	-0.052	*0.004*
explosive/aggressive	-0.036	*0.004*	0.021	*0.005*	0.342	*0.004*	-0.002	*0.004*
participates actively	0.267	*0.008*	0.224	*0.008*	0.077	*0.006*	-0.045	*0.004*
ask adults	**0.522**	*0.005*	0.152	*0.005*	-0.056	*0.003*	-0.003	*0.003*
interested in books	**0.604**	*0.004*	-0.076	*0.003*	0.025	*0.004*	0.146	*0.004*
interested in environment	**0.712**	*0.004*	0.040	*0.004*	-0.006	*0.002*	-0.046	*0.002*
plays to (dis)assemble	**0.569**	*0.005*	0.025	*0.004*	-0.035	*0.003*	-0.049	*0.003*
shows artistic interest	**0.519**	*0.005*	0.027	*0.004*	0.017	*0.004*	-0.021	*0.003*

Notes: RI-EFA estimates by maximum likelihood on panel data sample. Variables representing dedicated system in bold, standard error in italics.

S2 Appendix Fig 1. Distribution of random intercept in the measurement system

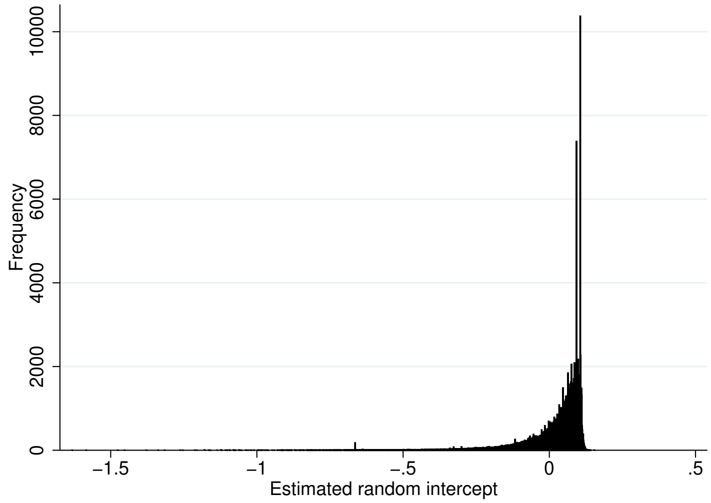

Supplement: S2 Appendix — (PDF) [file pone.0288186.s002.pdf]
