# Supplementary material for: Distributional effects of parental time investments on children’s socioemotional skills and nutritional health
Source: PLoS One. 2023 Oct 13;18(10):e0288186. doi: 10.1371/journal.pone.0288186 (PMC10575499; doi:10.1371/journal.pone.0288186)
Supplement: S3 Appendix — (PDF) [file pone.0288186.s003.pdf]

# S3 Appendix. Robustness checks

## Investment equation and production functions

This section reports different complementary analysis to understand reliability of the estimates of the production functions and the investment equations. S3 Appendix Table 1 reports standard specification tests to the investment equation estimates. I include the OLS population average estimates, fixed effects, mixed model and hybrid model (correlated random effects). S3 Appendix Table 2 shows the estimates of the production functions, without accounting or endogeneity on the parental investments. S3 Appendix Table 3 shows the estimates of the production functions, adjusting for attrition using the Inverse Propensity Weighing (IPW) method.

**S3 Appendix Table 1. Time investment equation: different specifications**

| | OLS (PA) | | FE | | Mixed | | Hybrid (CRE) | |
|---|---|---|---|---|---|---|---|---|
| Skills | **0.085** | *0.002* | **0.105** | *0.006* | **0.092** | *0.001* | **0.087** | *0.003* |
| Skills SE | | | | | **0.079** | *0.006* | **0.097** | *0.009* |
| BAZ | **0.003** | *0.001* | -0.001 | *0.010* | **0.002** | *0.001* | 0.002 | *0.010* |
| BAZ SE | | | | | **0.029** | *0.002* | 0.001 | *0.012* |
| First born | **0.050** | *0.004* | | | **0.043** | *0.002* | **0.040** | *0.003* |
| Exclusive breastfeeding | **0.052** | *0.003* | | | **0.044** | *0.000* | **0.044** | *0.000* |
| Number of siblings | **-0.049** | *0.002* | **-0.012** | *0.002* | **-0.045** | *0.001* | **-0.040** | *0.001* |
| Caretakers (number) | **0.029** | *0.002* | **0.015** | *0.002* | **0.025** | *0.000* | **0.027** | *0.000* |
| Ethnic background = 1 | **-0.051** | *0.007* | | | **-0.032** | *0.007* | **-0.032** | *0.007* |
| Household in urban area = 1 | **0.066** | *0.014* | | | **0.070** | *0.006* | **0.070** | *0.006* |
| Mother age at birth (log) | **-0.070** | *0.009* | | | **-0.075** | *0.000* | **-0.075** | *0.000* |
| Mother education (log years) | **0.026** | *0.004* | **0.012** | *0.004* | **0.024** | *0.001* | **0.030** | *0.001* |
| Father education (log years) | **0.025** | *0.002* | **0.006** | *0.002* | **0.017** | *0.000* | **0.020** | *0.001* |
| Mother in salary work = 1 | -0.002 | *0.004* | **-0.017** | *0.004* | -0.009 | *0.004* | -0.009 | *0.004* |
| Father in salary work = 1 | 0.008 | *0.005* | -0.003 | *0.004* | **0.010** | *0.005* | **0.010** | *0.005* |
| Mother self-employed = 1 | **0.021** | *0.004* | 0.006 | *0.004* | 0.001 | *0.004* | 0.001 | *0.004* |
| Father self-employed = 1 | **0.021** | *0.005* | 0.005 | *0.004* | **0.013** | *0.005* | **0.013** | *0.005* |
| Father figure present (Never) | | | | | | | | |
| Sometimes | **0.027** | *0.011* | 0.007 | *0.005* | **0.023** | *0.005* | **0.023** | *0.005* |
| Always | **0.146** | *0.005* | **0.054** | *0.004* | **0.111** | *0.005* | **0.111** | *0.005* |
| Pareting this child is (Easy) | | | | | | | | |
| Not easy nor hard | **-0.054** | 0.002 | **-0.018** | 0.003 | **-0.039** | 0.002 | **-0.033** | 0.002 |
| Hard | **-0.147** | 0.005 | **-0.045** | 0.006 | **-0.101** | 0.005 | **-0.082** | 0.005 |
| Pareting support (Always) | | | | | | | | |
| Sometimes | **-0.058** | 0.002 | **-0.028** | 0.003 | **-0.051** | 0.002 | **-0.041** | 0.002 |
| Never | **-0.074** | 0.004 | **-0.030** | 0.005 | **-0.062** | 0.004 | **-0.054** | 0.004 |
| Participation in social org. | **0.122** | *0.003* | **0.063** | *0.003* | **0.099** | *0.009* | **0.090** | *0.009* |
| School tuition (No pay) | | | | | | | | |
| $2 to $50 | **0.070** | *0.009* | | | **0.069** | *0.002* | **0.072** | *0.002* |
| $50 to $100 | **0.087** | *0.008* | | | **0.082** | *0.002* | **0.093** | *0.002* |
| $100 or more | **0.107** | *0.009* | | | **0.115** | *0.002* | **0.116** | *0.002* |
| School math z-score (grade 4) | **0.014** | *0.003* | | | **0.013** | *0.002* | **0.011** | *0.002* |
| School reading z-score (grade 2) | 0.005 | *0.003* | | | **0.007** | *0.008* | **0.017** | *0.008* |
| Home close to recreation area | **0.192** | *0.004* | **0.081** | *0.005* | **0.149** | *0.020* | **0.149** | *0.020* |
| Home close to public services | **0.078** | *0.006* | **0.035** | *0.005* | **0.057** | *0.011* | **0.057** | *0.011* |
| N | 178,220 | | 178,220 | | 178,220 | | 178,220 | |

Notes: significant values in bold (p<0.1). Standard errors in italics. PA: population average, CRE: correlated random effects.

**S3 Appendix Table 2. Production functions (exogenous time investments)**

| | Socioemotional (t+1) | | | | BMI (t+1) | | | |
|---|---|---|---|---|---|---|---|---|
| | Kindergarten | | 1st grade | | Kindergarten | | 1st grade | |
| | Boys | Girls | Boys | Girls | Boys | Girls | Boys | Girls |
| Investment | **0.02** | **0.02** | 0.01 | **0.01** | 0.01 | **-0.02** | -0.01 | **-0.02** |
| | *0.00* | *0.00* | *0.03* | *0.02* | *0.01* | *0.01* | *0.01* | *0.01* |
| BAZ | **-0.01** | **0.00** | **-0.01** | **-0.01** | **0.40** | **0.41** | **0.50** | **0.51** |
| | *0.00* | *0.00* | *0.00* | *0.00* | *0.01* | *0.01* | *0.01* | *0.01* |
| Socioemotional | **0.66** | **0.67** | **0.79** | **0.77** | **-0.04** | **-0.02** | **-0.03** | **-0.03** |
| | *0.01* | *0.01* | *0.01* | *0.01* | *0.01* | *0.02* | *0.01* | *0.01* |
| Mother education (log years) | **0.01** | -0.01 | **0.02** | **0.02** | -0.02 | **-0.03** | **-0.02** | 0.00 |
| | *0.00* | *0.00* | *0.01* | *0.01* | *0.01* | *0.01* | *0.01* | *0.01* |
| Father education (log years) | **0.01** | **0.01** | **0.01** | 0.00 | 0.00 | -0.02 | **-0.01** | 0.00 |
| | *0.00* | *0.00* | *0.00* | *0.00* | *0.01* | *0.01* | *0.01* | *0.01* |
| Mother's age at birth (log) | **0.12** | **0.12** | **0.15** | **0.12** | **0.08** | **0.07** | 0.03 | **0.06** |
| | *0.02* | *0.02* | *0.01* | *0.01* | *0.03* | *0.03* | *0.03* | *0.03* |
| Father figure present (Never=0) | | | | | | | | |
| Sometimes | -0.01 | 0.01 | 0.01 | 0.01 | 0.00 | -0.02 | -0.03 | -0.01 |
| | *0.01* | *0.01* | *0.01* | *0.02* | *0.02* | *0.02* | *0.05* | *0.02* |
| Always | **0.07** | **0.05** | **0.14** | 0.01 | -0.04 | -0.01 | 0.00 | -0.01 |
| | *0.01* | *0.01* | *0.02* | *0.02* | *0.02* | *0.02* | *0.05* | *0.02* |
| Age (log months) | 0.01 | 0.01 | **0.18** | **0.16** | **0.26** | **0.30** | **0.57** | **0.31** |
| | *0.02* | *0.02* | *0.05* | *0.04* | *0.09* | *0.08* | *0.03* | *0.07* |
| HAZ | 0.00 | 0.00 | 0.00 | 0.00 | **0.20** | **0.20** | **0.26** | **0.24** |
| | *0.00* | *0.00* | *0.00* | *0.00* | *0.01* | *0.01* | *0.01* | *0.01* |
| Weight at birth (log kg) | 0.00 | 0.00 | 0.02 | 0.01 | **0.41** | **0.34** | **0.36** | **0.37** |
| | *0.01* | *0.01* | *0.01* | *0.02* | *0.04* | *0.03* | *0.03* | *0.03* |
| Exclusive breastfeeding | **0.01** | 0.00 | **0.01** | **0.01** | **0.04** | 0.01 | **0.03** | **0.02** |
| | *0.00* | *0.00* | *0.01* | *0.01* | *0.01* | *0.01* | *0.01* | *0.01* |
| First born | **-0.01** | 0.00 | **0.04** | **0.03** | -0.01 | -0.02 | 0.01 | 0.00 |
| | *0.00* | *0.00* | *0.01* | *0.00* | *0.01* | *0.01* | *0.01* | *0.01* |
| Number of siblings | **-0.01** | 0.00 | **-0.02** | **-0.01** | **-0.04** | **-0.04** | **-0.03** | **-0.02** |
| | *0.00* | *0.00* | *0.00* | *0.00* | *0.01* | *0.01* | *0.01* | *0.01* |
| Ethnic background = 1 | 0.00 | 0.00 | **0.02** | **-0.02** | **0.11** | **0.04** | **0.10** | **0.07** |
| | *0.01* | *0.01* | *0.01* | *0.01* | *0.02* | *0.02* | *0.02* | *0.02* |
| Household in urban area = 1 | 0.01 | 0.01 | **0.02** | **-0.02** | -0.02 | -0.02 | -0.02 | -0.02 |
| | *0.01* | *0.01* | *0.01* | *0.01* | *0.02* | *0.02* | *0.02* | *0.02* |
| Adjusted R-squared | 0.48 | 0.46 | 0.47 | 0.46 | 0.18 | 0.19 | 0.30 | 0.31 |
| N | 45,661 | 46,680 | 45,522 | 48,572 | 45,661 | 46,680 | 45,522 | 48,572 |

Notes: significant values in bold (p<0.1). Standard errors in italics.

### S3 Appendix Table 3. Production functions (attrition analysis)

| | SED (t+1) | | | | BAZ (t+1) | | | |
|---|---|---|---|---|---|---|---|---|
| | Kindergarten | | 1st grade | | Kindergarten | | 1st grade | |
| | Boys | Girls | Boys | Girls | Boys | Girls | Boys | Girls |
| Investment | **0.13** | **0.08** | **0.07** | **0.06** | **-0.11** | **-0.05** | **-0.14** | **-0.13** |
| | *0.02* | *0.02* | *0.03* | *0.02* | *0.05* | *0.04* | *0.04* | *0.05* |
| BAZ | **-0.01** | **0.00** | -0.01 | **-0.01** | **0.37** | **0.38** | **0.46** | **0.47** |
| | *0.00* | *0.00* | *0.00* | *0.00* | *0.01* | *0.01* | *0.01* | *0.01* |
| SED | **0.67** | **0.66** | **0.78** | **0.75** | **-0.02** | -0.01 | **-0.01** | **-0.01** |
| | *0.01* | *0.01* | *0.01* | *0.01* | *0.01* | *0.01* | *0.01* | *0.01* |
| Mother education | **0.01** | 0.00 | **0.02** | **0.02** | -0.02 | -0.02 | -0.03 | -0.02 |
| | *0.00* | *0.00* | *0.01* | *0.01* | *0.01* | *0.01* | *0.01* | *0.01* |
| Father education | **0.01** | 0.01 | **0.01** | 0.00 | -0.01 | -0.01 | -0.01 | -0.01 |
| | *0.00* | *0.00* | *0.00* | *0.00* | *0.01* | *0.01* | *0.01* | *0.01* |
| Mother's age at birth (log) | **0.12** | **0.12** | **0.15** | **0.13** | **0.07** | **0.07** | 0.01 | **0.05** |
| | *0.01* | *0.02* | *0.01* | *0.01* | *0.03* | *0.03* | *0.03* | *0.03* |
| Father figure (Never) | | | | | | | | |
| Sometimes | 0.00 | 0.00 | 0.00 | 0.00 | 0.04 | -0.03 | -0.01 | 0.04 |
| | *0.01* | *0.01* | *0.01* | *0.01* | *0.01* | *0.03* | *0.02* | *0.01* |
| Always | **0.10** | **0.06** | **0.16** | **0.12** | **0.04** | -0.01 | -0.01 | **0.04** |
| | *0.01* | *0.01* | *0.02* | *0.01* | *0.02* | *0.04* | *0.03* | *0.02* |
| Age (log months) | **0.12** | 0.00 | **0.20** | **0.40** | **0.30** | **0.31** | **0.54** | **0.29** |
| | *0.02* | *0.02* | *0.05* | *0.04* | *0.07* | *0.08* | *0.03* | *0.07* |
| HAZ | 0.00 | **0.00** | 0.00 | 0.00 | **0.20** | **0.20** | **0.26** | **0.20** |
| | *0.00* | *0.00* | *0.00* | *0.00* | *0.01* | *0.01* | *0.01* | *0.01* |
| Weight at birth (log kg) | 0.01 | -0.02 | 0.01 | 0.01 | **0.34** | **0.31** | **0.20** | **0.30** |
| | *0.01* | *0.01* | *0.01* | *0.02* | *0.03* | *0.03* | *0.03* | *0.03* |
| Exclusive breastfeeding | **0.01** | 0.00 | **0.01** | **0.01** | **0.02** | 0.01 | **0.03** | **0.02** |
| | *0.00* | *0.00* | *0.01* | *0.01* | *0.01* | *0.01* | *0.01* | *0.01* |
| First born | **-0.01** | 0.00 | **0.02** | **0.02** | 0.01 | -0.01 | -0.01 | 0.01 |
| | *0.00* | *0.00* | *0.01* | *0.00* | *0.01* | *0.01* | *0.01* | *0.01* |
| Number of siblings | **-0.01** | **-0.01** | **-0.02** | **-0.01** | **-0.03** | **-0.04** | **-0.04** | **-0.03** |
| | *0.00* | *0.00* | *0.00* | *0.00* | *0.01* | *0.01* | *0.01* | *0.01* |
| Ethnic background = 1 | 0.00 | 0.00 | **0.02** | **-0.02** | **0.07** | **0.04** | **0.10** | **0.07** |
| | *0.01* | *0.01* | *0.01* | *0.01* | *0.02* | *0.02* | *0.02* | *0.02* |
| Household in urban area = 1 | 0.01 | 0.01 | **0.02** | **-0.02** | -0.02 | -0.02 | -0.02 | -0.02 |
| | *0.01* | *0.01* | *0.01* | *0.01* | *0.02* | *0.02* | *0.02* | *0.02* |
| Investment Res. | **-0.11** | **-0.07** | **-0.07** | **-0.04** | **0.13** | **0.05** | **0.16** | **0.12** |
| | *0.02* | *0.02* | *0.03* | *0.03* | *0.03* | *0.02* | *0.04* | *0.03* |
| Adjusted R-squared | 0.48 | 0.46 | 0.49 | 0.47 | 0.19 | 0.21 | 0.31 | 0.34 |
| N | 45,661 | 46,680 | 45,522 | 48,572 | 45,661 | 46,680 | 45,522 | 48,572 |

Notes: significant values in bold ($p<0.1$). Standard errors in italics.
