# Supplementary material for: Distributional effects of parental time investments on children’s socioemotional skills and nutritional health
Source: PLoS One. 2023 Oct 13;18(10):e0288186. doi: 10.1371/journal.pone.0288186 (PMC10575499; doi:10.1371/journal.pone.0288186)
Supplement: S4 Appendix — (PDF) [file pone.0288186.s004.pdf]

S4 Appendix. Heterogeneity on input complementarities

S4 Appendix Fig 1 shows the effects on future human capital, in standard deviations, from increasing BAZ or socioemotional development one standard deviation at each decile of the socioemotional development baseline distribution. The top-left graph shows the large persistence of socioemotional development, higher at lower SED levels, consistent with previous evidence for non-cognitive and cognitive socioemotional development atta2.

Similarly, the prevalence of BAZ is substantial and increasing between grades through the distribution (bottom-right panel). The inverted U-shape is consistent with larger yearly variation in BAZ among underweight and obese children. Consistent with evidence of developing and developed countries, higher persistence in first grade can be attributed mainly to natural physiological changes around age six, known as adiposity rebound. Children who rebound younger are also more likely to be obese, which also explains the broader effect of age on BAZ in first grade, where is more likely for a larger proportion of children to experience the inflection point in BAZ.

Evidence of dynamic complementarities between SED and BAZ is relatively stable and weak. In the bottom-left panel, most estimates are not statistically different from zero, except for boys in the middle of the BAZ distribution in Kindergarten. For obese children there does not seem to be any significant association either in a given year or by sex. The effect of BAZ on socioemotional development is presented in the top-right panel. There is a positive effect of roughly 0.1 SD in SED from reducing BAZ by one SD among children at the bottom of the SED distribution. Given the relative distance between children with healthy weight and those who are severely obese, gains in socioemotional development from a large reduction in BAZ among severely obese children (roughly 6% of all students) could be approximately 0.3 SD in a year.

Fig 5. S4 Appendix Fig 1. Marginal product of socioemotional skills and nutritional health

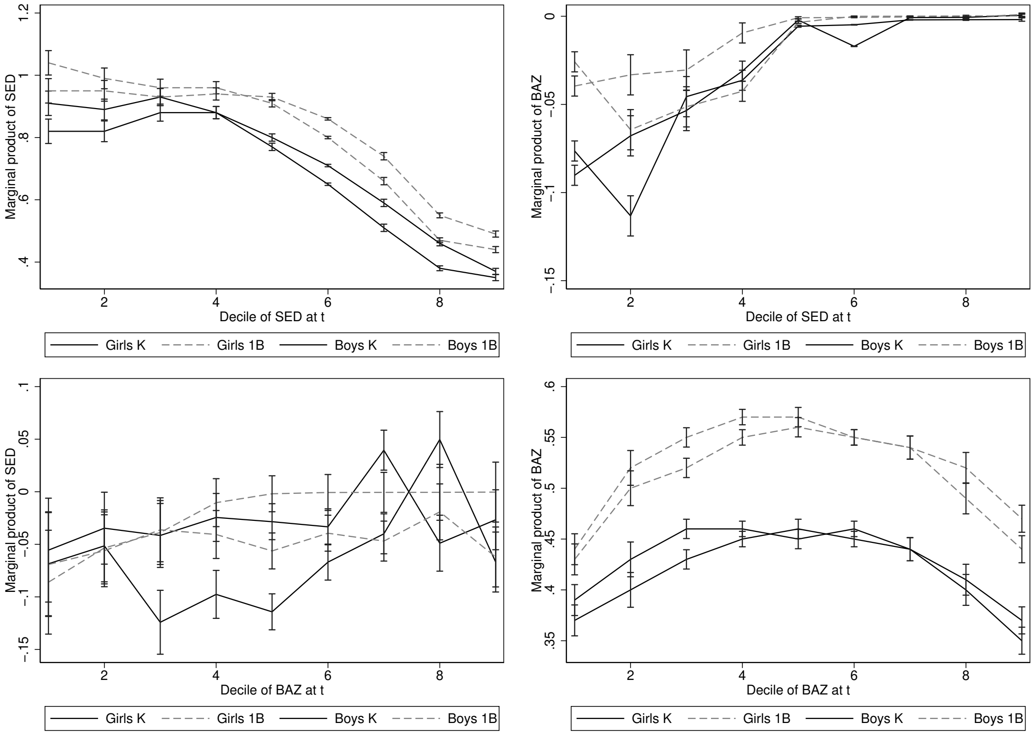

Notes: Vulnerability deciles are constructed based on the school vulnerability index (IVE). Calculations based on the longitudinal matched JUNAEB data. Latent scales are constructed, so log means are zero.
