# Supplementary material for: Distributional effects of parental time investments on children’s socioemotional skills and nutritional health
Source: PLoS One. 2023 Oct 13;18(10):e0288186. doi: 10.1371/journal.pone.0288186 (PMC10575499; doi:10.1371/journal.pone.0288186)
Supplement: Attachment — Submitted filename: PONE-D-23-03208_reviewer comments.pdf [file pone.0288186.s005.pdf]

Distributional effects of parental time investments on children's socioemotional skills and nutritional health

PONE-D-23-03208

Reviewer report

Comments to author

First, I would like to point out to the author that I found their paper quite interesting and enjoyed reading it.

However, I would like to give some recommendations that may contribute to improve this work.

1. I have missed an explanation of why the author has selected this country and not another one for this paper.

2. I consider that the data used for this paper are correct, but it should be mentioned as a limitation of this paper that there is no information on private schools. Hofflinger et al. (2020) show in Chile that a key assumption of school choice and competition policies is that parents' most important (if not only) priority in choosing a school is its quality. Chile is a country with a national system of school choice and competition and in this sense the authors in their study find that parents who choose a school prioritize its proximity, its quality and whether it provides religious education. In their results they show that the probability that parents prioritize proximity is higher for parents of low socioeconomic status, while the probability that they prioritize religious education and quality is higher for parents of high socioeconomic status. Their findings show that only advantaged families choose schools based on their quality and, therefore, school choice and competition policies may offer limited benefit for disadvantaged students, possibly maintaining or reinforcing socioeconomic segregation in the educational system.
   It would be necessary to mention this data limitation in the paper.

3. It would be important to mention and go a little deeper into the paper, that there are different types of child care. The literature shows that it is important to classify the type of child care, and some authors classify it into three types: basic, educational, and supervisory child care (Guryan et al., 2008; Gimenez-Nadal and Molina, 2012; Campaña et al., 2017). Within these three activities mentioned above, the activities aimed at increasing the human capital of children are those found in educational child care. And regarding differences between fathers and mothers: Fathers prefer childcare activities that are more rewarding, such as playing with their children or helping them with homework (Craig, 2006a; Darling-Fisher & Tiedje, 1990; Giménez-Nadal & Molina, 2013; Grossman, Pollack, & Golding, 1988; Kahneman & Krueger, 2006; Kahneman, Krueger, & Schkade, 2004).

4. It is important to delve a little deeper into the following: The existing literature clearly shows that parents with high levels of education tend to spend more time with their children, compared with parents with low levels of education (Bianchi, Cohen, Raley, & Nomaguchi, 2004; Campaña et al., 2017; Craig, 2006b; Gimenez-Nadal & Molina, 2013; Guryan, Hurst, & Kearney, 2008; Hofferth, 2001; Marsiglio, 1991; Sayer, Bianchi, & Robinson, 2004; Sayer et al., 2004) partly due to the fact that more educated parents recognise and acknowledge the importance of time investments in their children (Kalenkoski & Foster, 2008; Marsiglio, 1991; Sayer et al., 2004; Sevilla & Borra, 2015). Better educated parents spend more time in educational childcare activities, including reading, playing, and helping with homework (Gimenez-Nadal & Molina, 2013; Hill & Stafford, 1985; Kalenkoski & Foster, 2008; Sayer et al., 2004), that promote the development of human capital of children (Brooks-Gunn et al., 2002).

5. It is feasible to include in the analysis a variable indicating the presence or number of other household members (other than the father, mother or younger siblings of the children analyzed). Delgado and Canabal (2006) and Fuller, Holloway, and Liang (1996) show that members of Hispanic families allocate their time according to their strong family orientation (focusing on the family group), and parents tend to use other family members to care for their children.

6. Why are individuals' wages and non-labor income not taken into account in the explanatory variables? Research has shown that higher wages lead to a better position at home as it increases the bargaining power within the couple (Bourguignon, Browning, & Chiappori, 2009; Chiappori, 1988, 1992; Lundberg & Pollak, 1993), and this higher bargaining power may be used to take responsibility for more rewarding childcare activities, such as playing with or reading to children. Non-labour income may also affect the time parents devote to childcare. Kalenkoski, Ribar, and Stratton (2005) show that mothers reduce the time devoted to active childcare when household income increases.

7. It would be feasible to include a dummy variable of whether the children analyzed live in an urban or rural area. The female labor participation rate is lower in rural areas than in urban areas. In addition, there are other control variables, such as indigenous population and family structure, which can vary considerably from one area to another.

8. Regarding mothers' work, it is feasible to know whether they are self-employed or salaried. The literature shows a positive relationship between self-employment and time spent on childcare (Conelly 1992; Edwards and Field-Hendrey 1996; Caputo and Dolinsky 1998; Boden 1999; Gimenez et al., 2013; Campaña et al., 2020).
