# Supplementary material for: Distributional effects of parental time investments on children’s socioemotional skills and nutritional health
Source: PLoS One. 2023 Oct 13;18(10):e0288186. doi: 10.1371/journal.pone.0288186 (PMC10575499; doi:10.1371/journal.pone.0288186)
Supplement: Attachment — Submitted filename: PONE-D-23-03208-R1-Reviewer 2 comments.pdf [file pone.0288186.s007.pdf]

Distributional effects of parental time investments on children's socioemotional skills and nutritional health

PONE-D-23-03208-R1

Reviewer #2

Comments

The author has correctly answered my concerns, and he has made changes in the paper considering my suggestions.

In my opinion, I would recommend the paper for publication in the Plos One journal.
